# Bioaccumulation of radionuclides in hoofed animals inhabiting the Semipalatinsk Test Site

**DOI:** 10.1371/journal.pone.0294632

**Published:** 2023-11-27

**Authors:** Andrey Panitskiy, Asem Bazarbaeva, Symbat Baigazy, Yelena Polivkina, Ivan Alexandrovich, Mariya Abisheva

**Affiliations:** Institute of Radiation Safety and Ecology, NNC RK, Kurchatov, Kazakhstan; University of South Carolina, UNITED STATES

## Abstract

The article assesses the content of radionuclides in hoofed animals inhabiting the Semipalatinsk Test Site by calculation. Hoofed animals’ faeces were sampled to determine the content of radionuclides in their diets. Based on values determined for the content of radionuclides in animals; diets, the content of radionuclides in the meat and milk of farm animals—cows (*Bos taurus taurus*), sheep (*Ovis*), goats (*Capra hircus*) and horses (*Equus caballus Lin*., 1758) as well as in the meat of wild animals–european moose (*Alces alces Lin*., 1758), argali (*Ovis ammon Lin*., 1758), roe deer (*Capreolus pygargus Pal*., 1771) and saiga (*Saiga tatarica Lin*., 1766) was calculated. No excess of permissible values of the content of ^137^Cs and ^90^Sr in the meat of farm animals was found to be expected, even for a conventional ‘conservative’ scenario, in which maxima of the radionuclide activity concentration in a vegetable feed (faeces) are taken as a basis. ^241^Am and ^239+240^Pu in the meat of farm hoofed animals are not standardized. Their predicted maxima of activity concentration are very low, and even in the ‘conservative’ scenario, they do not exceed 1.8×10^−2^ Bq kg^-1^, 1.4×10^−1^ Bq kg^-1^ and 1.6×10^−1^ Bq kg^-1^, respectively. In the milk of farm animals, the content of ^137^Cs and ^90^Sr does not exceed permissible values. ^241^Am and ^239+240^Pu in the milk of farm animals are not standardized. Their predicted activity concentration values in the milk of sheep and goats do not exceed 6.5×10^−2^ Bq l^-1^, for cows– 2.6×10^−2^ Bq l^-1^, for horses– 3.1×10^−2^ Bq l^-1^. Permissible values of ^137^Cs and ^90^Sr in the meat of wild hoofed animals are not exceeded either. In the meat of argali, roe deer and saigas, relatively high levels of ^137^Cs are predictable. ^241^Am and ^239+240^Pu in meat of wild animals are not standardized. Their predicted activity concentration values in the meat of moose and argali do not exceed 3.2×10^−1^ Bq kg^-1^ and 1.6×10^−1^ Bq kg^-1^, respectively, for roe deer and saiga—5.4×10^−2^ Bq kg^-1^. Thus, in case of free grazing in the STS territory, no excess of permissible values of standardized radionuclides (^137^Cs and ^90^Sr) in the meat and milk of hoofed animals is predictable.

## Introduction

**Fig 1:**
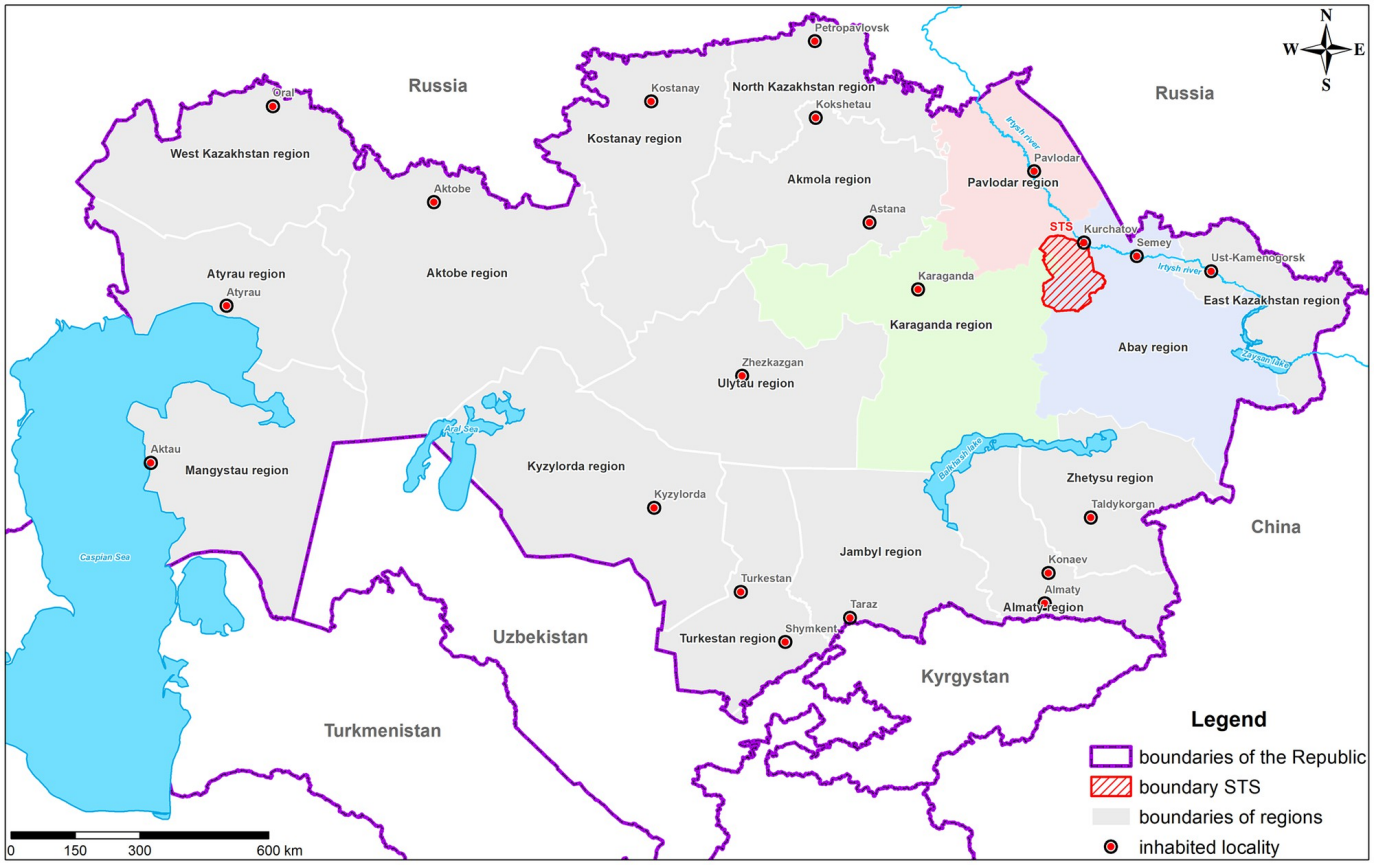
STS schematic location in three regions of the Republic of Kazakhstan.

Between 1949 and 1991, nuclear tests were conducted in the Republic of Kazakhstan (the former Kazakh SSR) in the territory of the Semipalatinsk Test Site (STS). This 18 thous. square kilometer test site covers three modern regions in the Republic of Kazakhstan–the Pavlodar region, the Karaganda region and the Abai region (the former Semipalatinsk region) (Fig 1).

Over the years of testing, 486 nuclear tests were conducted at the test site (atmospheric (aboveground and air), underground, excavation (with soil ejected)) [1], which resulted in environmental contamination. That said, ecosystems became contaminated both directly at nuclear test locations (‘Experimental Field’, ‘Balapan’, ‘Degelen’, ‘Sary-Uzen’, ‘4’, ‘4a’ sites, objects ‘Atomic Lake’, ‘Telkem-1’ and ‘Telkem-2’) and beyond–due to the fallout in the form plumes [2–10]. The fallout in the form of plumes produced by aboveground nuclear tests on September 24, 1951 and August 12, 1953 impacted areas outside test locations. The areas where tests were directly conducted will remain controlled by the state for a long time due to the complicated radioecological situation. Issues to release a portion of test site areas outside test locations are consistently raised by farmers living in areas adjacent to STS. Despite bans, farmers are engaged in unauthorized grazing of farm animals and procure plant forage (mowing steppe motley grasses). In addition, the STS territory is inhabited by animals that are subject to amateur and commercial hunting including big hoofed animals–european moose (*Alces alces* Lin., 1758), roe deer (*Capreolus pygargus* Pal., 1771), saiga (*Saiga tatarica* Lin., 1766) and argali (*Ovis ammon* Lin., 1758) that is red-listed by the Republic of Kazakhstan and the International Union for Conservation of Nature. Activity concentrations of man-made radionuclides were determined in some animal products (meat, milk) from individual farms that carry out unauthorized economic activities [11,12]. However, it is impossible to approximate the results for the entire STS territory since its area is large and nonuniformly contaminated with radionuclides. It does not seem possible to sample products from each farm at STS because those who work at these farms either do not wish to interact with researchers or are just hired workers. Therefore, as part of a comprehensive environmental survey of the STS territory, the possible content of man-made ^137^Cs, ^90^Sr, ^239+240^Pu and ^241^Am was assessed in animal products if produced within STS using a calculation technique. This calculation technique for determining activity concentrations of radionuclides in the muscle tissue and milk of animals is based upon the measurement of activity concentrations of radionuclides in faeces of big hoofed animals sampled in the STS territory on the assumption that activity concentrations of radionuclides in animals’ faeces correspond to the ones in the diet of a particular animal [13]. The meat of wild hoofed animals inhabiting the STS territory was also similarly evaluated since, currently, there has been a transition from the anthropocentric model of radiation protection to the environmental protection [14,15].

Thus, the purpose of research with results quoted in the article is to assess the possible content of man-made radionuclides in the meat and milk of big hoofed animals inhabiting STS areas outside nuclear weapon test locations and which are potentially releasable to the economic use.

## Materials and methods

### Field activities

**Fig 2:**
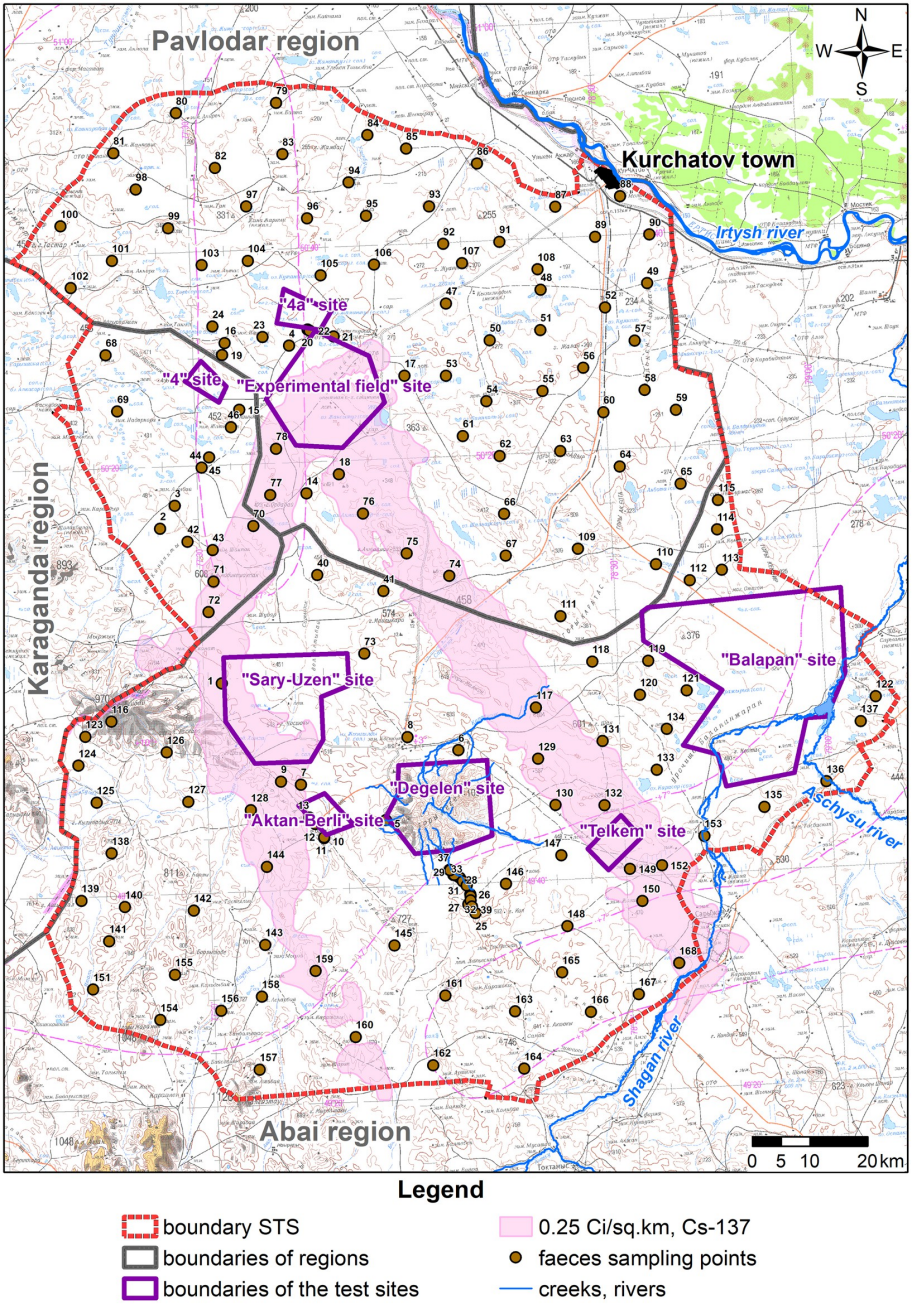
Schematic sampling of big hoofed animals’ faeces.

By big hoofed animals the paper implies farm animals such as cows (*Bos taurus taurus*), sheep (*Ovis*), goats (*Capra hircus*), horses (*Equus caballus* Lin., 1758) and wild animals–european moose (*Alces alces* Gray, 1821), argalis (*Ovis ammon* Lin., 1758), roe deer (*Capreolus pygargus* Pal., 1771) and saigas (*Saiga tatarica* Lin., 1766). The main way for farm animals to feed at STS is to graze freely in natural pastures. At the same time, our observations showed that animals graze in this region all the year round. This is due to low precipitation in the form of snowfall in the winter season in this region. Wild animals are also freely feeding in natural pastures of the test site. Thus, any specific sample from these animals’ faeces may characterize animals’ daily diet that may contain radionuclides. That said, faeces contain activities of radionuclides that are produced by all the major potential sources of intake–plant forage, water and soil that animals may accidentally ingest when feeding. To determine activity concentrations of radionuclides in diets of big hoofed animals, 168 samples of animal faeces were collected (both from wild and farm animals freely grazing)–roe deer, saigas, argalis, cows, horses, sheep and goats. Sampling was on a one-sample-per-100-km^2^ basis. Samples were collected over a relatively uniform grid in the survey area. Sampling some of faeces that contacted the soil cover was not allowed. STS faeces sampling points were topographically mapped with isolines of fallout plumes (contamination density with cesium-137) from aboveground tests on September 24, 1951 and August 12, 1953 (Fig 2). Both ‘plumes’ were identified during the aerial gamma-spectrometric survey in 1990 and 1991.

### Laboratory research

#### Radionuclide analysis

Faeces samples, prior to the measurement, were previously dried at a room temperature, charred at 400 °C and sieved through a 0.1 mm mesh (in order to homogenize the sample). Activity concentrations of ^137^Cs and ^241^Am in dry faeces samples were determined in charred samples with a γ-spectrometer Canberra GX-2020 in a plastic container shaped like a 94 mm straight cylinder. The measurement time was at least 60 min. The minimum detectable activity (MDA) for ^137^Cs and ^241^Am was 0.1 Bq kg^-1^ and 0.06 Bq kg^-1^. To calibrate the γ-spectrometer, calibration sources IAEA-RGK-1 Potassium Sulfate, IaEa-RGTh-1 Torium Ore, IAEA-RGU-1 Uranium Ore diluted were used. Measurements were performed as per the gamma-spectrometer procedure [16].

Once measured with a gamma spectrometer, samples were additionally radiochemically prepared [17]. ^90^Sr activity was determined from that of ^90^Y accumulated with an ultralow-background liquid-scintillation spectrometer-radiometer Quantulus 1220 using Cherenkov radiation. The chemical yield of Sr and Y carriers was determined with an atomic emission spectrometer [18]. MDA for ^90^Sr was 0.6 Bq kg^-1^. To calibrate by ICP-AS, multi-element solutions of reference samples (RS) containing metals were used, manufactured by Inorganic Ventures IV-ICP-MS-71A with a certified nominal value of metal content equal to 10 mg L^-1^ and the uncertainty of a certified value equal to 0.5% (dilution factor, k = 2).

^239+240^Pu activity concentration was measured with an alpha-spectrometer having a solid-state detector ‘Alpha-Analyst’ (‘CANBERRA’, USA) [18]. MDA for ^239+240^Pu was 0.01 Bq kg^-1^.

#### Mapping

Maps quoted in the article were constructed using the ArcGIS application. Digitized maps of Kazakhstan were acquired by the branch ‘Institute of Radiation Safety and Ecology’ of the Republican State Enterprise on REM ‘National Nuclear Center of the Republic of Kazakhstan’ from the Republican State Public Enterprise ‘National Map-Making and Geodetic Fund’ of the Geodesy and Mapping Committee of the Ministry of Digital Development, Innovations and Aerospace Industry of the Republic of Kazakhstan under state procurement contract No. 02-19/122 dated 28.04.2020.

#### Quality control

Research was undertaken using the analytical and test equipment calibrated and tested as per the Law of the Republic of Kazakhstan dated June 7, 2000 No. 53-II ‘On assurance of the uniformity of measurements’.

For the purpose of measurement quality control, in the course of determining radionuclide activity concentrations in faeces samples, one ‘replicate’ sample was added to each batch of test faeces samples. This sample was intended for quality control and repeatability of analytical results. To control a hypothetical cross-contamination, a ‘reference’ sample was also added to the batch. The ‘replicate’ test sample was randomly chosen from the set in the batch. The ‘reference’ sample was prepared from plants with a known content of radionuclides to be analyzed at the ‘background’ level. The test and ‘reference’ samples were analyzed simultaneously with all of the other samples.

### Processing of results

The most common hoofed animals were assessed on the survey land (cows (*Bos taurus taurus*), sheep (*Ovis*), goats (*Capra hircus*), horses (*Equus caballus* Lin., 1758)). Such animal products as meat and milk including the meat of european moose (*Alces alces* Lin., 1758), argali (*Ovis ammon* Lin., 1758), roe deer (*Capreolus pygargus* Pal., 1771) and saiga (*Saiga tatarica* Lin., 1766) were addressed.

The calculation technique to determine a potential activity concentration in products of animal origin is based upon the measurement of activity concentrations of radionuclides in an animal’s faeces followed by conversion to the ones in an animal’s muscular tissue was applied as per the formula:

$${\text{A}}_{\text{m},\text{i},\text{prod}}=\text{DMI}\times {\text{A}}_{\text{m},\text{i},\text{for}}\times {\text{F}}_{\text{f}}{}_{,\text{m}}$$ (1)

DMI–(dry matter intake)–a daily dry matter intake (forage intake), kg d^-1^;

A_m,i,for_−the activity concentration of the i^th^ radionuclide in the forage (faeces) of herbivores, Bq kg^-1^;

F_f,m_−the transfer factor of the i^th^ radionuclide from the forage to products (muscular tissue (F_f_) or milk (F_f,m_)).

Transfer factors of a radionuclide for products were taken from the IAEA document–TRS-472 (F_f_−‘Feed transfer coefficient’) F_f_−the mass or volumetric activity density in the receptor animal tissue or animal product (in Bq kg^-1^ of fresh weight or Bq l^-1^ for milk) divided by the daily intake of a radionuclide (in Bq d^-1^). Unit–d kg^-1^ or d l^-1^, where d is the time in days [19].

The table (Table 1) lists mean values of transfer factors for radionuclides (F_f_) in mutton, beef, horse beef and cow milk (F_m_) according to TRS-472 [19]. No F_f_ values were derived for the meat of roe deer, moose and saiga, therefore F_f_ for similar farm animals–cows, goats and sheep were used. In particular, cow F_f_ values were used for moose, sheep values for argali, goat F_f_ were used for roe deer and saiga. No F_f_ or F_m_ were detected for horse meat and milk. Therefore, F_f_ and F_m_, values derived for cows were applied in the assessment. Transfer factors of ^241^Am and ^239+240^Pu for goat meat and that of ^239+240^Pu for goat milk were also missing. Therefore, transfer factor values derived for sheep were used for these types of products. Transfer factors of ^241^Am were also missing for sheep meat, therefore a transfer factor obtained for goat meat was used. Thus, the table (Table 1) lists transfer factors of radionuclides from animals’ diets to products and these were used in further assessment.

**Table 1:** Transfer factors (F_f_ or F_m_) of radionuclides for animal products [1,6].

Type of product	^137^Cs	^241^Am	^239+240^Pu	^90^Sr
Sheep and argali meat	1.9×10^−1^	1.1×10^−4^	5.3×10^−5^	1.5×10^−3^
Goat, roe deer and saiga meat	3.2×10^−1^	6.9×10^−6^	5.3×10^−5^	2.9×10^−3^
Cow and moose meat	2.2×10^−2^	5.0×10^−4^	1.1×10^−6^	1.3×10^−3^
Horse meat	2.2×10^−2^	5.0×10^−4^	1.1×10^−6^	1.3×10^−3^
Cow milk	4.6×10^−3^	4.2×10^−7^	1.0×10^−5^	1.3×10^−3^
Horse milk	4.6×10^−3^	4.2×10^−7^	1.0×10^−5^	1.3×10^−3^
Sheep milk	5.8×10^−2^	6.9×10^−6^	1.0×10^−4^	2.7×10^−2^
Goat milk	1.1×10^−1^	6.9×10^−6^	1.0×10^−4^	1.6×10^−2^

To calculate activity concentrations of radionuclides in animal products, the daily forage intake (DMI) by sheep and goats was taken equal to 2 kg d^-1^, the one by cows– 15 kg d^-1^, by horses– 18 kg d^-1^ [20–23]. According to literature sources, an adult moose is able to consume up to 11 kg per day in the wintertime and 25 to 35 kg of a dry plant forage in the summertime, an argali—about 18 kg, a roe deer– 2 kg and more in the wintertime and up to 6 kg in the summer time, a saiga– 3 to 6 kg [24–26]. Thus, the daily forage intake (DMI) by moose and argali is taken equal to 35 kg d^-1^ and 18 kg d^-1^, respectively. For Siberian roe deer and saiga this value is 6 kg d^-1^.

When calculating activity concentrations of radionuclides in products (A_m,i,prod_), activity concentration values of each radionuclide in faeces of big hoofed animals reported from radionuclide analyses were taken as the activity concentration of the i^th^ radionuclide in the forage of big hoofed animals (A_m,i,feed_). To that end, data was statistically processed using the STATISTICA 12 software package. Previously, activity concentration values of radionuclides below MPA were removed from the retrieval in question.

## Results and discussion

Value ranges of activity concentrations of radionuclides for big hoofed animals’ faeces are listed below (Table 2).

**Table 2:** Activity concentrations of radionuclides in big hoofed animals’ faeces, Bq kg^-1^.

Radionuclides	Activity concentrations										
	N	Mean	GM	*M*_*e*_	*M*_*o*_	Min	Max	Range	SD	CV	SE
^137^Cs	106	3.4	2.5	2.5	multiple	0.4	26.0	25.6	3.6	107.2	0.4
^241^Am	30	2.0	0.9	0.7	multiple	0.07	12.0	11.9	2.9	144.7	0.5
^90^Sr	128	17.0	7.9	7.4	7.5	0.6	380.0	379.4	43.5	256.0	3.9
^239+240^Pu	97	2.7	0.7	0.6	0.3	0.04	71.1	71.1	8.7	322.8	0.9

**Fig 3:**
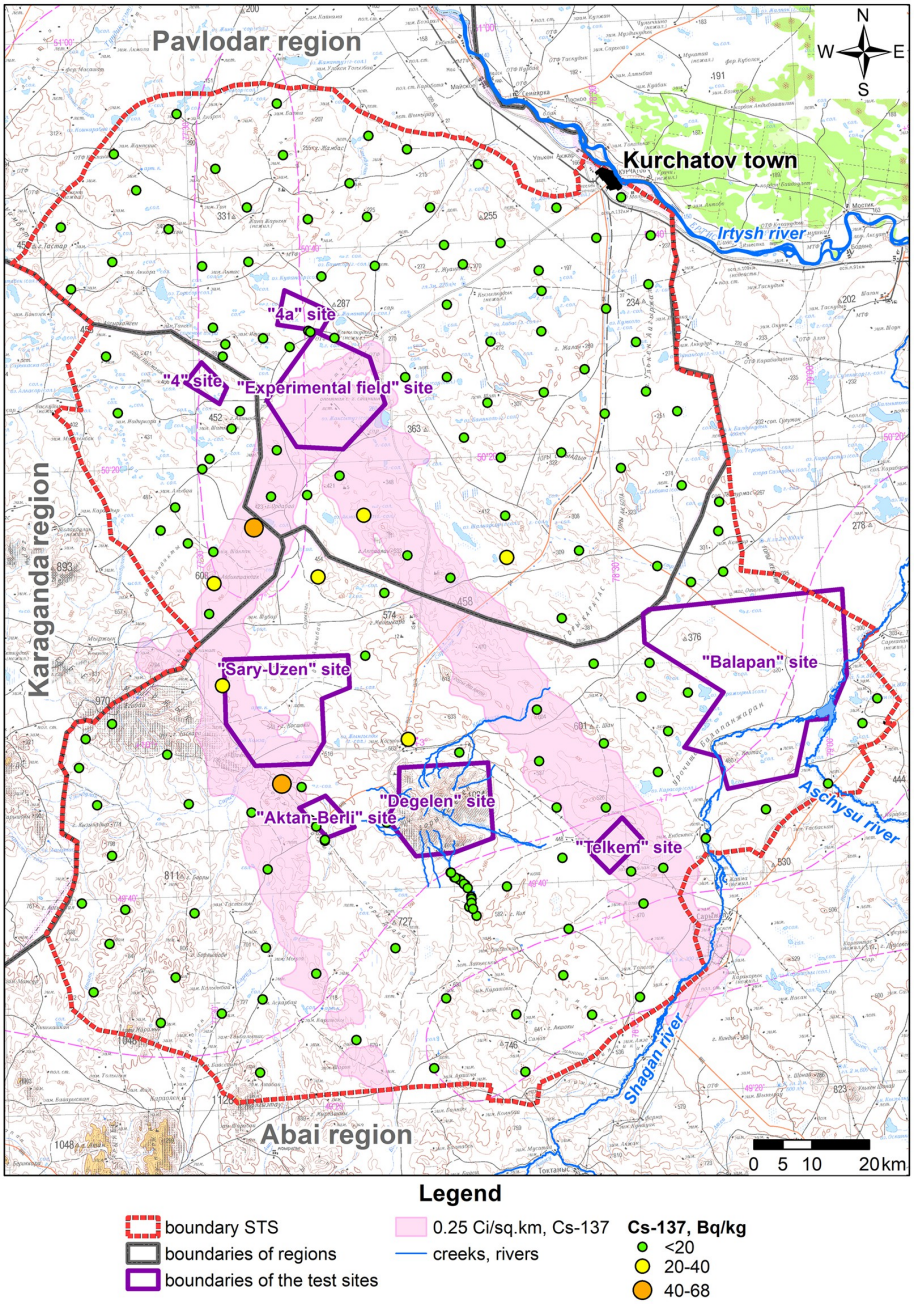
Distribution of ^137^Cs activity concentration in big hoofed animals’ faeces.

**Fig 4:**
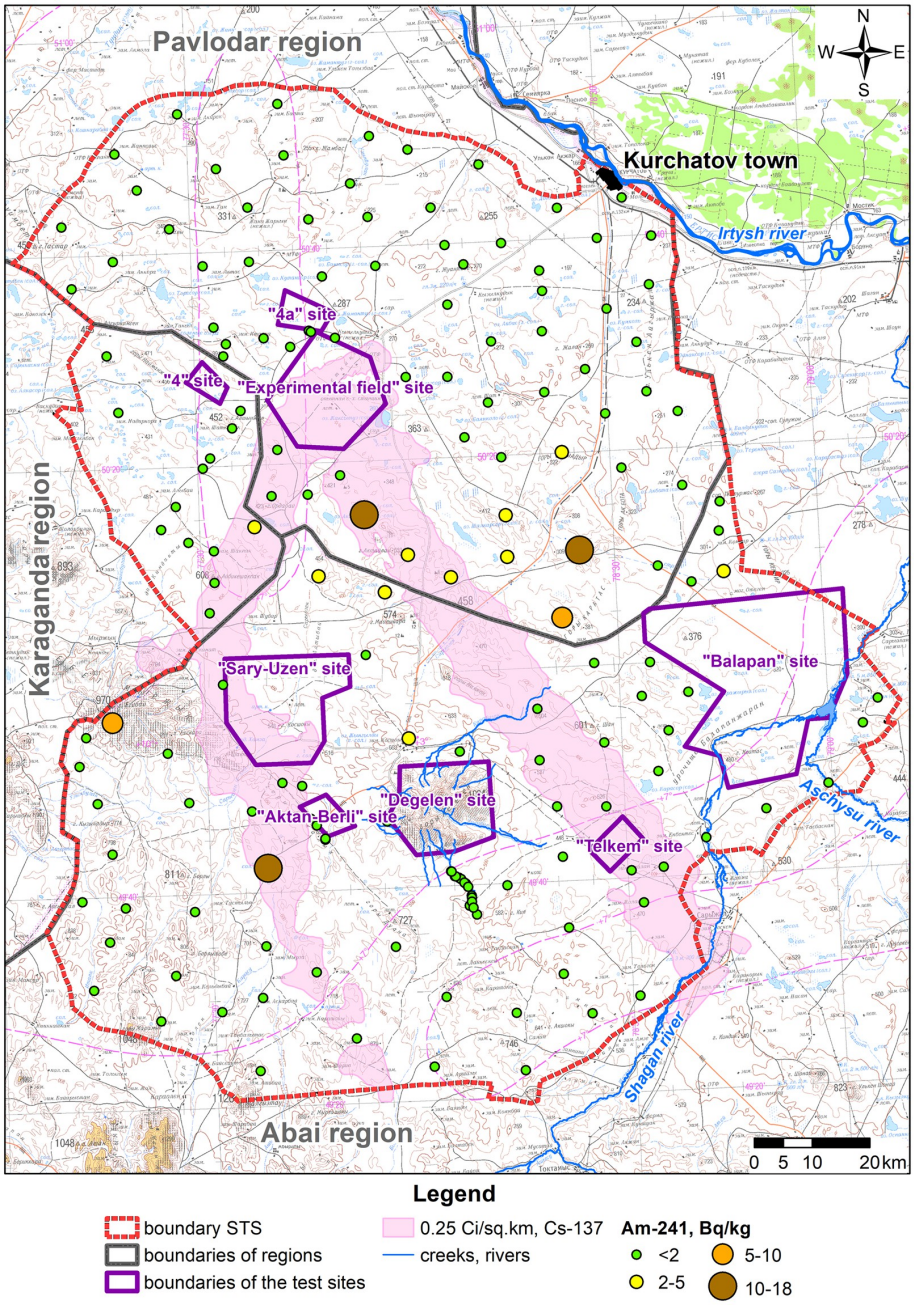
Distribution of ^241^Am activity concentration in big hoofed animals’ faeces.

**Fig 5:**
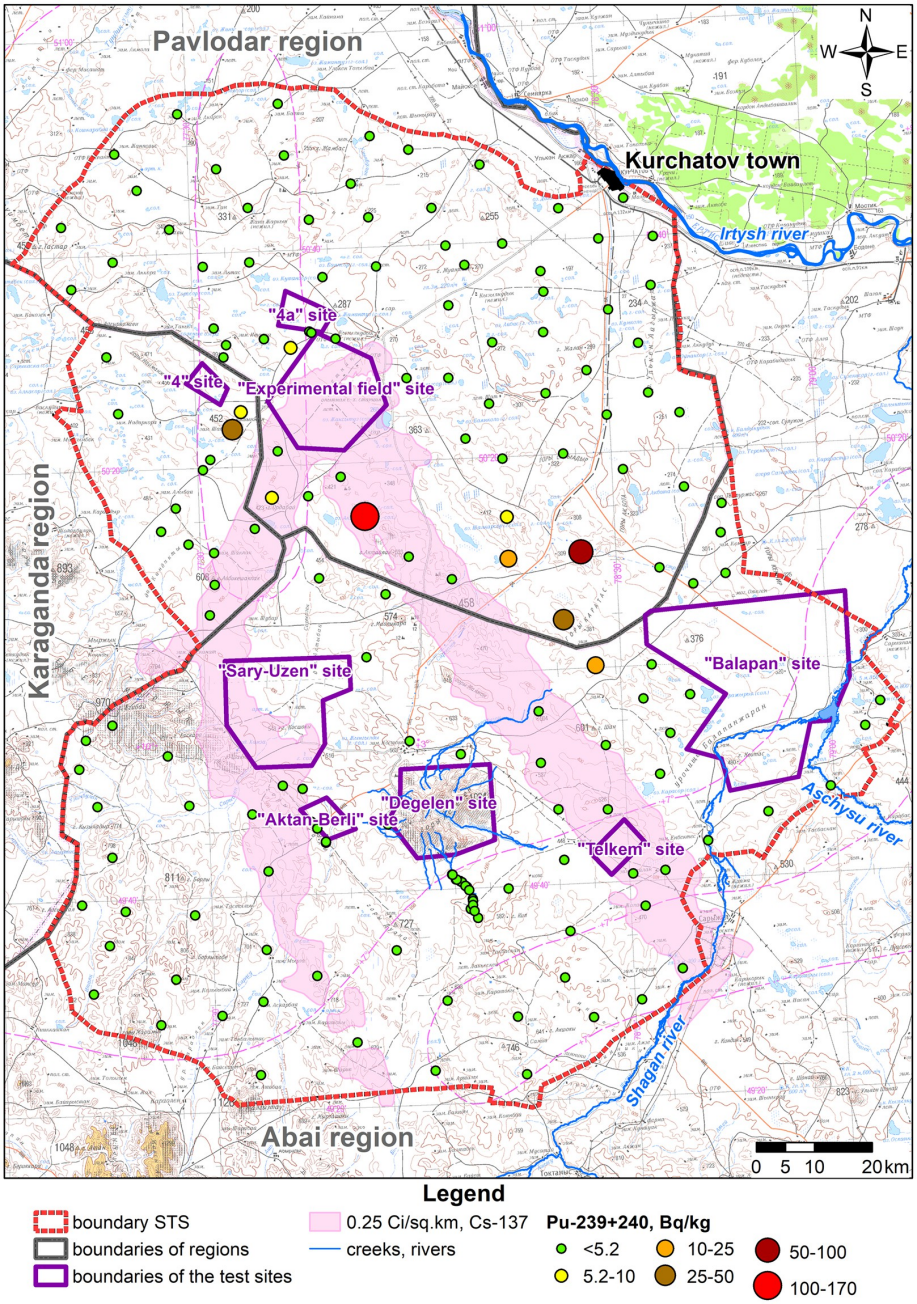
Distribution of ^239+240^Pu activity concentration in big hoofed animals’ faeces.

**Fig 6:**
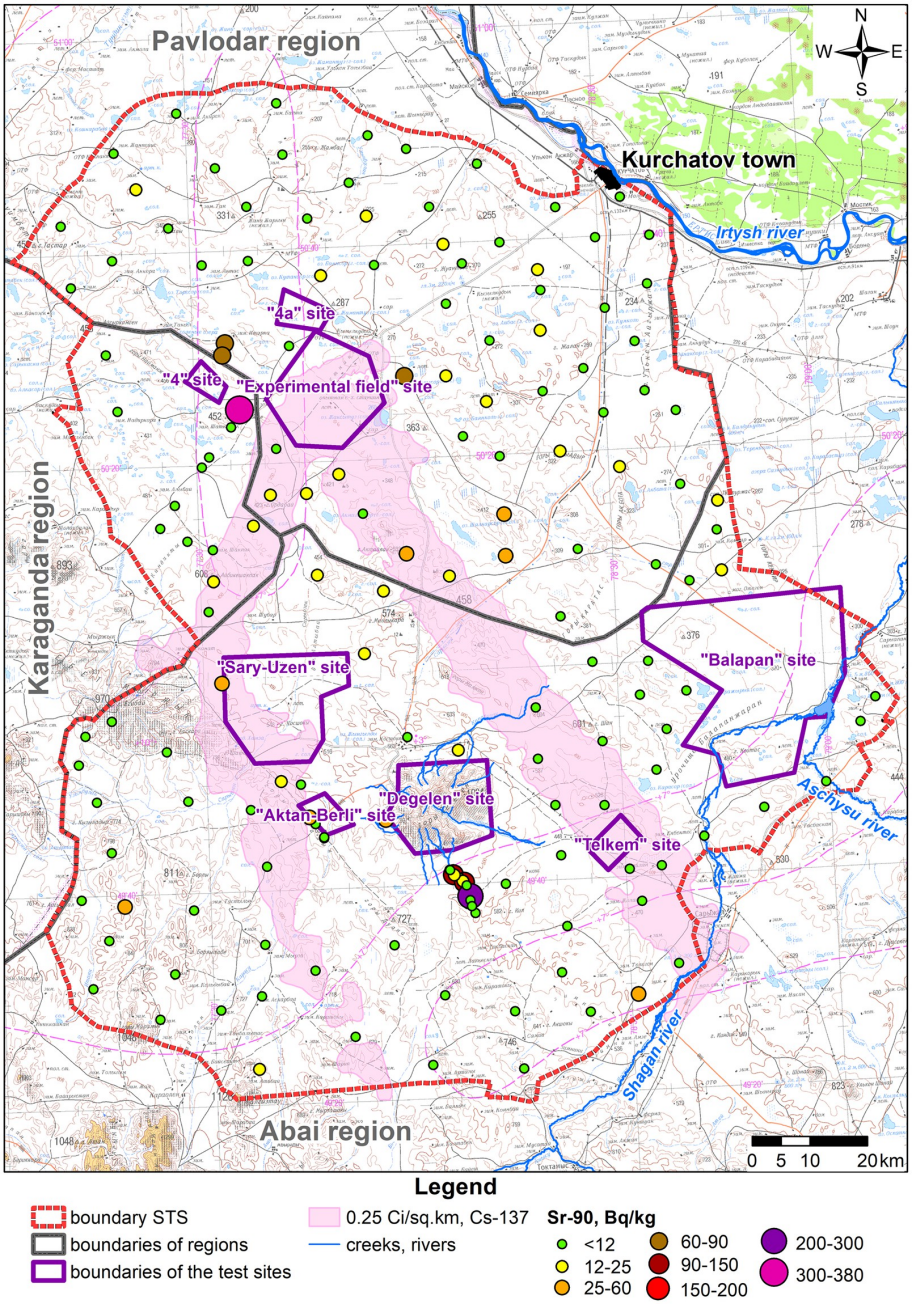
Distribution of ^90^Sr activity concentration in big hoofed animals’ faeces.

Due to a wide variation, the statistical population of data under consideration is heterogeneous, and the mean value is atypical and cannot be used as a general indicator of this population. Therefore, when calculating radionuclide activity concentration in products (A_m,i,prod_), the maximum activity concentration value of each radionuclide in faeces of big hoofed animals was taken as that of the i^th^ radionuclide in the forage of big hoofed animals (A_m,i,feed_). Distributions of radionuclide activity concentrations in faeces depending on a faeces sampling location are depicted in figures below (Figs 3–6).

The highest values of ^137^Cs activity concentration in big hoofed animals’ faeces are noted for the fallout plume area as a result of the 1951 test (Fig 3).

Values of ^241^Am activity concentrations are the highest for fallout plumes that resulted from the 1951 and 1953 tests. That said, mean values of ^241^Am activity concentration for areas mentioned are the same (Fig 4). Overall, no high values of ^239+240^Pu activity concentration are noted in faeces. Its highest recorded activity concentration value does not exceed 18 Bq kg^-1^. This may be due to poor migration properties of this isotope and, first of all, in the food chain ‘soil–plants’ [27].

The highest value of ^239+240^Pu activity concentration is noted in the fallout plume from the 1953 test (Fig 5). Elevated values of ^239+240^Pu are also noted along the plume mentioned.

Mean values of ^90^Sr activity concentration in all of the areas in question are quite close but the highest value was recorded in the conventionally ‘background’ STS territory (Fig 6). This is attributable to the fact that maxima of ^90^Sr activity concentration were recorded near the ‘4A’ test location (Fig 6), in which radiological warfare agents were tested and high values of ^90^Sr activity concentration are recorded in the soil and plants (up to n×10^8^ Bq kg^-1^) [27,28]. Elevated values of ^90^Sr are also recorded in faeces samples collected from final creek runoffs at the Degelen mountain range (the ‘Degelen’ site), at which underground nuclear weapon tests were conducted in adits from which radionuclides have been carried away through creeks to this day [29].

It is clear from the data quoted that the presence of radionuclides in the plant forage of wild hoofed animals is associated with areas of radioactively contaminated environmental components (fallout in the form of plumes, sections close to test locations or fallout plumes). The presence of elevated values of radionuclides in animals’ faeces noted in conventionally ‘background’ areas illustrates that animals can move freely around areas considered in the paper when grazing. Therefore, radionuclide activity concentrations were calculated for the entire STS territory using activity concentration maxima of radionuclides in faeces. Such more conservative approach eliminates the risk of underestimating the potential hazard to products obtained from hoofed animals inhabiting STS. Values listed in the table (Table 1) are taken as F_f_ values (for milk–F_m_) (Feed transfer coefficient). Results calculated for the content of radionuclides in the meat of farm animals are tabulated below (Table 3).

**Table 3:** Activity concentrations of radionuclides in the meat of farm animals, Bq kg^-1^.

Type of product	^137^Cs	^241^Am	^239+240^Pu	^90^Sr
Permissible level [30]	200	not standardized	not standardized	50
Sheep meat	26	4.0×10^−3^	1.8×10^−2^	1.1
Goat meat	44	2.5×10^−4^	1.8×10^−2^	2.2
Cow meat	22	1.4×10^−1^	2.8×10^−3^	7.4
Horse meat	27	1.6×10^−1^	3.4×10^−3^	8.9

According to findings, the excess of permissible values for the content of ^137^Cs and ^90^Sr in the meat of farm animals is not predicted, even in the ‘conservative’ assessment, which is based on the maxima of radionuclide activity concentrations in the vegetable forage (faeces) and which is unlikely in case of freely grazing animals due to their constant movement. ^241^Am and ^239+240^Pu in the meat of hoofed farm animals are not standardized. Their predicted activity concentration maxima are very low, and even with a ‘conservative’ assessment they do not exceed 1.8×10^−2^ Bq kg^-1^, 1.4×10^−1^ Bq kg^-1^ and 1.6×10^−1^ Bq kg^-1^ for the meat of sheep and goats, cows, horses, respectively.

The table (Table 4) lists the results calculated for content of radionuclides in the milk of farm hoofed animals.

**Table 4:** Activity concentrations of radionuclides in the milk of hoofed farm animals, Bq l^-1^.

Type of product	^137^Cs	^241^Am	^239+240^Pu	^90^Sr
Permissible level [30]	100	not standardized	not standardized	25
Cow milk	4.7	1.1×10^−4^	2.6×10^−2^	7.4
Horse milk	5.6	1.4×10^−4^	3.1×10^−2^	8.9
Sheep milk	7.9	2.5×10^−4^	3.4×10^−2^	21.0
Goat milk	15	2.5×10^−4^	6.5×10^−2^	12

As in the case of meat, no excess of permissible values of the content of ^137^Cs and ^90^Sr is expected in the milk of farm animals in any of the areas in question. ^241^Am and ^239+240^Pu in the milk of farm animals are not standardized. Their predicted activity concentration values for sheep and goat milk do not exceed 6.5×10^−2^ Bq l^-1^, for cow milk– 2.6×10^−2^ Bq l^-1^, for horse milk– 3.1×10^−2^ Bq l^-1^.

The table (Table 5) lists results calculated for the content of radionuclides in the meat of wild hoofed animals.

**Table 5:** Activity concentrations of radionuclides in the meat of wild hoofed animals, Bq kg^-1^.

Animal species	^137^Cs	^241^Am	^239+240^Pu	^90^Sr
Permissible level [30]	300	not standardized	not standardized	100
Moose	52	3.2×10^−1^	6.5×10^−3^	17
Argali	230	3.6×10^−2^	1.6×10^−1^	10
Roe deer, saiga	130	1.2×10^−2^	5.4×10^−2^	3.4

As in the case of farm animals, no excess of permissible values of ^137^Cs and ^90^Sr is expected in the meat of wild hoofed animals. Relatively elevated values of ^137^Cs are predicted in the meat of argali, roe deer and saiga. ^241^Am and ^239+240^Pu in meat of wild animals are not standardized. Their calculated activity concentration values in the meat of moose and argali do not exceed 3.2×10^−1^ Bq kg^-1^ and 1.6×10^−1^ Bq kg^-1^, roe deer and saiga– 5.4×10^−2^ Bq kg^-1^.

As a whole, results have demonstrated that no higher than normal activity concentration values of radionuclides considered are predicted in hoofed animals inhabiting the STS territory despite that they freely move around test locations. For comparison–mean activity concentrations of ^137^Cs in the meat of mooses that inhabits the exclusion and evacuation zone over the entire observation period following the Chernobyl accident were 9.2×10^3^ Bq kg^-1^ and 3.3×10^3^ Bq kg^-1^, respectively [31]. Mean values of ^137^Cs activity concentrations in the roe deer in the exclusion zone were 1.7×10^4^ Bq kg^-1^, and in the evacuation zone– 6.8×10^3^ Bq kg^-1^ [32]. Higher values of ^137^Cs activity concentration in the meat of wild animals in these zones may be associated with the ones in environmental components of these areas rather than in most of the STS territory where tests were not directly conducted. This may also be due to the ability of ^137^Cs to produce fixed forms over time, and more than 70 years have passed since the main tests were conducted, which had impacted the progression of the radioecological situation at STS most of all. For example, in STS soils, irrespective of the origin of radioactive contamination, ^137^Cs is characterized by a low mobility. It is mostly contained in soils in a tightly bound form—from 93% (at the ‘4a’ RWA test location) to 96% (in areas affected by radioactive water streams (the ‘Degelen’ site) and in the global fallout (conventionally background areas)) and 99% at objects contaminated with the fallout from aboveground and excavation blasts (‘Experimental Field’ site, ‘Atomic Lake’ objects). [9,33]. ^90^Sr activity concentration in the muscular tissue and internal organs of wild hoofed animals varied between 30 and 110 Bq kg^-1^ for the exclusion zone and between 11 and 30 Bq kg^-1^ –for the evacuation zone [34]. Thus, activity concentrations of these radionuclides in the muscle tissue of hoofed animals inhabiting STS are much lower than the ones in the muscle tissue of hoofed animals inhabiting individual areas contaminated with radionuclides as a result of the Chernobyl radiation incident.

Thus, the assessment of content of radionuclides in hoofed animals inhabiting the Semipalatinsk Test Site showed that elevated values of man-made radionuclides in faeces of hoofed farm and wild animals are recorded not only in areas of radioactively contaminated environmental components (fallout in the form of plumes) but also in conventionally ‘background’ STS areas that were not exposed to higher than normal contamination with radioactive isotopes in nuclear weapon tests. This is attributed to the fact that while feeding animals move around STS areas of different environmental radioactivity levels (in the soil, plants and water). Farm and wild hoofed animals move around these areas. Despite this, none of the areas in question is expected to exceed permissible values for the content of radionuclides in the meat and milk of farm animals and meat of wild animals, even with a ‘conservative’ assessment, whereby activity concentration maxima of radionuclides in animals’ diets (calculated from the data on the content of radionuclides in faeces of these animals) were taken as a basis. Such ‘conservative’ scenario is unlikely in case animals are freely grazing due to their constant movement and the pattern of radioactive contamination in the STS territory in the form of local radioactive areas (‘spots’). Relatively elevated values of ^137^Cs are expectable in the meat of argali, roe deer and saiga. However, due to the fact that activity concentration values were derived from the most conservative one, one can state that in case of freely moving animals around different STS areas, values of ^137^Cs activity concentration in the meat of these wild animals will be far lower. At the same time, it is necessary to take into account that elevated values were only reported for ^137^Cs, and its content in environmental components (constituting animals’ diets) can be expected to decrease given its half-life, which is 30.2 years [35].

The calculation results of radionuclide activity concentrations in animal products are in good agreement with direct measurements of radionuclide activity concentrations in animal products obtained at STS [11,12] Values reported can be used to initially evaluate radiation risks for biota, the contribution by foodstuffs to the ionizing radiation dose in case man lives in STS areas potentially releasable to the economic turnover, to make decisions regarding the possible economic or recreational utilization of these areas. Findings are also important for planning research both within STS and in other areas of a complicated radiological situation.

## Conclusions

Based upon values reported for the content of radionuclides in animals’ diets (from radionuclide activity concentrations in their faeces) inhabiting the STS territory, the content of radionuclides in the meat and milk of farm and wild animals (cows (*Bos taurus taurus*), sheep (*Ovis*), goats (*Capra hircus*), horses (*Equus caballus Lin*., 1758, european moose (*Alces alces Lin*., 1758), argali (*Ovis ammon Lin*., 1758), roe deer (*Capreolus pygargus Pal*., 1771) and saigas (*Saiga tatarica Lin*., 1766) was calculated. No excess of permissible values for the content of ^137^Cs and ^90^Sr in the meat and milk of farm and wild hoofed animals was found. ^241^Am and ^239+240^Pu in the meat and milk are not standardized. Their predicted activity concentration maxima do not exceed 1.8×10^−2^ Bq kg^-1^, 1.4×10^−1^ Bq kg^-1^ and 1.6×10^−1^ Bq kg^-1^ for the meat of sheep and goats, cows, horses, respectively. In sheep and goats’ milk, activity concentration values of ^241^Am and ^239+240^Pu do not exceed 6.5×10^−2^ Bq l^-1^, in cow’s milk– 2.6×10^−2^ Bq l^-1^, in mare’s milk– 3.1×10^−2^ Bq l^-1^. In the meat of argali, roe deer and saigas, a relatively elevated content of ^137^Cs is predicted. Activity concentration values of ^241^Am and ^239+240^Pu in the meat of moose and argali do not exceed 3.2×10^−1^ Bq kg^-1^ and 1.6×10^−1^ Bq kg^-1^, respectively, for roe deer and saiga– 5.4×10^−2^ Bq kg^-1^. Thus, in case of free grazing in the STS territory, no excess of permissible values of standardized radionuclides (^137^Cs and ^90^Sr) in the meat and milk of hoofed animals is predicted.
